# Missed opportunities for HIV testing and sexual health-related challenges in an individual with intellectual disability: a case report

**DOI:** 10.1186/s12981-024-00606-7

**Published:** 2024-04-05

**Authors:** Lina Martina Würfel, Anja Potthoff, Sandeep Nambiar, Adriane Skaletz-Rorowski

**Affiliations:** 1 WIR-Walk In Ruhr – Center for Sexual Health and Medicine, Große Beckstraße 12, 44787 Bochum, Germany; 2 Interdisciplinary Immunological Outpatient Clinic, Center for Sexual Health and Medicine, Department of Dermatology, Venereology and Allergology, Ruhr-Universität Bochum, Bochum, Germany

**Keywords:** Stigma, Intellectual disability, HIV, Sexuality, HIV testing, Public health

## Abstract

**Background:**

HIV testing remains an important tool in identifying people living with HIV/AIDS (PLWHA). An early diagnosis of HIV can lead to a prolonged life expectancy if treatment is initiated promptly. Indicator conditions can be the first sign of an HIV infection and should therefore be recognised and consequently a HIV test should be carried out. Testing should occur in all individuals as sexuality can be experienced by everyone, and stigma can lead to the exclusion of vulnerable groups, leading to a gap in diagnosis and treatment [1, 2].

**Case presentation:**

A 63-year-old man, who identifies as bisexual and has had an intellectual disability since birth, presented at our health care centre for HIV testing. A decade ago, the patient was diagnosed with Stage III Diffuse Large B-cell Non-Hodgkin Lymphoma, an AIDS defining cancer. The patient presented at a Haematology and Oncology department 3 months prior, due to a weight loss of 10 kg over the past 5 months. Oral thrush, an HIV-indicator condition, had been diagnosed by the otolaryngologists shortly before. During this medical evaluation, pancytopenia was identified. Despite the presence of indicator conditions, the patient was never tested for HIV in the past. Staff members from the care facility for intellectually disabled suggested conducting a HIV test in our clinic through the public health department, where HIV positivity was revealed. The AIDS-defining diagnosis, along with a CD4 + cell count of 41/µl, suggests a prolonged period of HIV positivity.

**Conclusion:**

Due to the presence of existing indicator conditions, an earlier HIV diagnosis was possible. We contend that most of the recent illnesses could have been prevented if earlier testing had been carried out. Therefore, patients presenting with AIDS indicator conditions, including those with mental disabilities, should be given the opportunity to be tested for HIV. HIV/AIDS trainings should be made available to health care professionals as well as to personnel interacting with vulnerable groups.

## Background

HIV testing continues to be a crucial method for identifying people living with HIV/AIDS (PLWHA) [3]. An early detection of HIV, followed by prompt initiation of treatment, can contribute to an extended life expectancy. Recognizing indicator conditions as potential early signs of HIV infection is essential, underscoring the importance of promptly conducting an HIV test. HIV-indicator conditions are those associated with or as a result of immunodeficiency and include AIDS-defining conditions [2, 4]. Testing should be inclusive, as everyone, regardless of their sexual orientation and their mental capacity, can be vulnerable to HIV.

## Case presentation

A 63-year-old bisexual man, with an intellectual disability since birth, presented at our center for HIV testing. Ten years prior, he had been diagnosed with Diffuse Large B-cell Non-Hodgkin Lymphoma in Stage III, an AIDS-defining cancer that requires an HIV Test [5]. Subsequently, he underwent therapy with Rituximab (8x) and CHOP (cyclophosphamide, doxorubicin hydrochloride (hydroxydaunomycin), vincristine sulfate (Oncovin) and prednisone) (6x). Three months before attending our center, he presented at a department of Hematology, Oncology, and Palliative Medicine for further investigation due to pancytopenia and a weight loss of 10 kg over the last 5 months (BMI: 17.6 kg/m2). At that time, the patient also reported experiencing heartburn. The patient denied having fever, chills, and night sweats. Additionally, there were recurrent middle ear infections, with the most recent one resulting in a perforated eardrum. Furthermore, there was an increase in episodes of panic attacks and the possibility of epilepsy was evaluated. The otolaryngologists had diagnosed oral thrush, a HIV-indicator condition, and the patient had already been receiving treatment with Amphotericin B suspension.

Amongst the diagnostics that were carried out to further investigate the symptoms the patient was presenting were, a CT scan, an esophagogastroduodenoscopy, and a bone marrow biopsy. The CT scan of the neck and thorax revealed a persistently stable lymphadenopathy, with some additional regression; a recurrence of Diffuse Large B-cell Non-Hodgkin Lymphoma could therefore be excluded. The CT scan showed an incidental finding of a hepatosplenomegaly and 4 small nodules up to 8 mm in the right lung. The esophagogastroduodenoscopy only revealed scars and transverse furrows of unclear etiology, with no evidence of a sustained fungal infection. Amphotericin B suspension was subsequently discontinued. A bone marrow biopsy was also performed but yielded no significant findings. The patient was subsequently released from the hospital. An HIV test was not carried out.

Following a training session on HIV, employees from the care facility for disabled individuals suggested carrying out an HIV test in our patient. Testing was carried out in our clinic in collaboration with the public health department, which led to the identification of HIV positivity.

The highest viral load was 73,763 copies/ml with a CD4 + helper cell count of 41/µl. The reduced CD4 + helper cell count, and the history of AIDS-defining and HIV-associated diseases imply that the diagnosis of HIV had been delayed for an extended period.

At the time of presentation at our medical center, there were no indications for other sexually transmitted infections.

Upon conversation with the patient, it emerged that he has resided in a residential facility for individuals with intellectual disabilities since 1996, with his legal caregiver being his brother. In terms of his sexual history, he was in a heterosexual relationship for eight years in the past. In 2011, the patient established a stable relationship with a homosexual man. Following this, he engaged in regular sexual encounters with different partners both inside and outside the facility. There is no record of drug use, and condom usage was infrequent.

## Discussion

Given the patient’s HIV-indicator conditions among the medical history such as oral thrush, pancytopenia, and wasting syndrome, as well as Diffuse Large B-cell Non-Hodgkin Lymphoma, an AIDS-defining cancer diagnosed in 2013, the question now arises as to when the HIV infection may have occurred and whether an earlier diagnosis would have been possible if HIV testing had been carried out, since indicator conditions were present.

Possible barriers hindering a timely diagnosis may have been: Stigma among physicians, which could entail erroneous assumptions regarding the sexuality of individuals with mental disabilities, failure to identify indicator conditions and test for HIV, lack of awareness among affected individuals with mental disabilities, resulting in a limited understanding of HIV and potential omission of crucial information during medical consultations, the failure to acknowledge sexuality and inadequate collection of sexual history, and insufficient inclusivity in HIV testing for all individuals.

The estimated median time for seroconversion to a CD4 + cell count below 200 cells/mm3 lies at 7,93 years [6], although not definitive, there is a strong likelihood that HIV would have been detected if HIV testing had been conducted at the onset of an indicator condition, a decade earlier. Furthermore, early HIV treatment initiation would likely have mitigated a significant part, if not all, of the more recent illness and probable HIV-related complications. This highlights the significance of HIV testing. 

Assumptions and stereotyping may lead a physician to wrongly believe that a mentally disabled person is incapable of engaging in sexual relations [7].

Consequently, this stereotyping could undermine the patient’s diagnosis, impede treatment, and hinder the attainment of positive health outcomes [8]. It is crucial to diagnose HIV early in order to initiate treatment promptly, as PLWHA who start highly active antiretroviral therapy (HAART) at a later stage, with a lower CD4 + cell count, seem to exhibit a higher propensity for AIDS-related complications at advanced ages, in contrast to those who initiated treatment earlier [9].

Provider-initiated testing for indicator conditions may hold particular significance for individuals with intellectual disabilities, as it could hinder their comprehension of HIV, their ability to disclose risky behavior, and/or their capacity to seek testing independently.

On the other hand, individuals at risk of acquiring HIV and PLWHA frequently experience elevated rates of mental health issues in comparison to the general population. Therefore, it is of great importance to integrate diagnostic methods such as HIV tests among the routine checkups to reduce the impact of stigma. HIV Testing should be inclusive for everyone, regardless of the social status, disabilities, and living conditions, sexuality should be addressed openly and assumptions should be avoided [10, 11].

Providing HIV/AIDS training through workshops and trainings for healthcare workers and related personnel who interact with vulnerable groups has proven to be highly significant, as demonstrated in this instance. If the center for disabled individuals had not proposed an HIV test for our patient, the diagnosis might not have been uncovered [12].

Additionally, sexuality needs to be acknowledged and addressed in individuals with disabilities, including those with learning disabilities, to provide education on safer sex practices and to facilitate HIV testing.

Overall, medical teams failed to recommend HIV testing in this patient multiple times, initially during the 2013 lymphoma diagnosis and at subsequent presentations with symptoms suggestive of AIDS. However, care facility staff deserve credit for recognizing the necessity of HIV testing after training and ensuring its arrangement.

## Conclusions

Patients presenting with indicator conditions, including those with mental disabilities, should be tested for HIV to ensure an early diagnosis, and all patients should be asked about their sexuality as everyone can be vulnerable to HIV. Furthermore, more trainings should be made available to health care professionals and related personnel regarding sexual health.

## Data Availability

No datasets were generated or analysed during the current study.
